# Severe skin disease in lupus associated with hemophagocytic lymphohistiocytosis: case reports and review of the literature

**DOI:** 10.1186/s41927-019-0055-x

**Published:** 2019-02-08

**Authors:** Christina S. Thornton, Parham Minoo, Michelle Schneider, Aurore Fifi-Mah

**Affiliations:** 1 Department of Medicine, University of Calgary, Calgary, Alberta Canada; 2 Department of Pathology and Laboratory Medicine, University of Calgary, Calgary, Alberta Canada; 3 Division of Rheumatology, Department of Medicine, University of Calgary, Calgary, Alberta Canada; 4 Rheumatology Outpatient Clinic, South Health Campus, 4448 Front Street SE, Calgary, Alberta T2M 1M4 Canada

## Abstract

**Background:**

Hemophagocytic lymphohistiocytosis (HLH) is a severe clinical entity associated with high mortality in the adult population. HLH has been associated with infections, malignancy and autoimmune conditions such as Systemic Lupus Erythematosus (SLE), however this is often in the context of a disease flare. Currently, there are limited reports of inaugural SLE manifesting as HLH with a lack of consensus on treatment and management of these patients.

**Case presentation:**

Here, we present two rare case reports of severe cutaneous manifestation of lupus associated with HLH. Both patients presented with sinister clinical courses with primarily rheumatologic complaints including malaise, arthralgia, and myalgia with biochemical abnormalities. Both patients were diagnosed with HLH as a result of first presentation from cutaneous lupus. A comprehensive literature review using the PubMed database with cases comprising keywords of HLH and SLE up to September 2017 was conducted, with an emphasis on inaugural cutaneous SLE cases.

**Conclusions:**

Ultimately, we highlight that a keen clinical acumen is required as misdiagnosis may lead to insufficient treatment with adverse clinical outcomes with the unique presentation of HLH from inaugural cases of SLE.

## Background

Hemophagocytic lymphohistiocytosis (HLH) is a constellation of symptoms caused by dysregulated hyperinflammation and cytokine storm, resulting in a life-threatening syndrome. HLH is classified into primary (familial) and secondary etiologies (infection, autoimmune conditions, drugs and malignancy) [1]. Often, HLH is associated with pediatric rheumatic conditions, however there is a growing body of literature reporting HLH in the older population.

Clinically and biochemically, the hallmark features include hepatosplenomegaly, fever, hyperferritinemia, hypofibrinogenemia, hypertriglyceridemia and pancytopenia [2]. While the true incidence of HLH is unknown, the mortality if left untreated is high and often patients succumb in days to months from multi-organ failure. However, with rapid identification of HLH and initiation of treatment, the survival rate approaches 50% [3, 4].

Given the main barrier in treatment is delay in diagnosis; HLH requires keen clinical acuity in order to attenuate complications from the sequela of disease. While there have been several reports in the literature of HLH in adulthood associated with autoimmune conditions such as systemic lupus erythematosus (SLE), often these have been associated with disease flares. We present here two cases of cutaneous lupus as the initial manifestation of HLH, a rare but clinically relevant entity. Accurate diagnosis is critical as the therapeutic approach may differ depending on the severity of HLH manifestation [5, 6].

## Case presentation

A 73-year-old Caucasian male presented to our acute care tertiary hospital with a several day history of rash that initially started on the scalp and was felt to be due to sunburn from outdoor exposure, but subsequently spread over the torso and arms with associated blistering. He also began to develop increasing fatigue and malaise, which prompted him to seek medical attention. His past medical history was significant only for hypertension and osteoarthritis. He denied any medications but did acknowledge alcohol substance use disorder. Remainder of review of systems was otherwise unremarkable.

On admission, he was febrile at 38.9 °C, heart rate was 110 beats/min, blood pressure was 105/82 and respiratory rate was 18 breaths/min. Physical examination was significant for skin findings including multiple flaccid bullae on an erythematous base with serosanguinous fluid diffusely over the torso, back and arms. A thick confluent plaque over the scalp was also noted. Palpable purpura at the lower extremities was present with petechiae to the fingers and toes. There was no mucosal involvement. The remainder of physical examination including precordium, respiratory and abdomen were within normal limits.

Initial laboratory investigations revealed pancytopenia (hemoglobin: 105 g/L; platelets: 53 × 10^9^/L, white blood cell: 3.3 × 10^9^/L,), CRP of 19.1 mg/L (0–8 mg/L) and ESR of 28 mm (0–10 mm). Haptoglobin was low at 0.09 g/L (0.3–2.0 g/L), suggesting an element of hemolysis. Albumin was low at 23 g/L (33–48 g/L) lactate dehydrogenase was increased at 349 U/L (100–235 U/L), as well as alanine aminotransferase at 141 U/L (1–40 U/L) and gamma glutamyl-transferase at 201 U/L (11–63 U/L). Ferritin was profoundly elevated at > 8000 μg/L (13–150 μg/L). Fibrinogen and D-dimer were within normal limits. Triglycerides were mildly elevated at 2.04 mmol/L (0.0–1.70 mmol/L). An initial immunological work-up showed an ANA titre of 1:80 with a homogenous and speckled pattern. ENA revealed positive Smith and RNP antibody. C3 and C4 were both depressed at 0.32 g/L (0.6–1.6 g/L) and 0.04 g/L (0.1–0.4 g/L), respectively. Of note, atypical ANCA was observed on indirect immunofluorescence but MPO and PR3 ANCA by ELISA were negative. Soluble IL-2R by ALBIA (addressable laser bead immunoassay methodology) was high. Abdominal ultrasound demonstrated heavy hepatic steatosis but no evidence of hepatosplenomegaly. Chest X-ray and echocardiogram were within normal parameters.

**Fig. 1:**
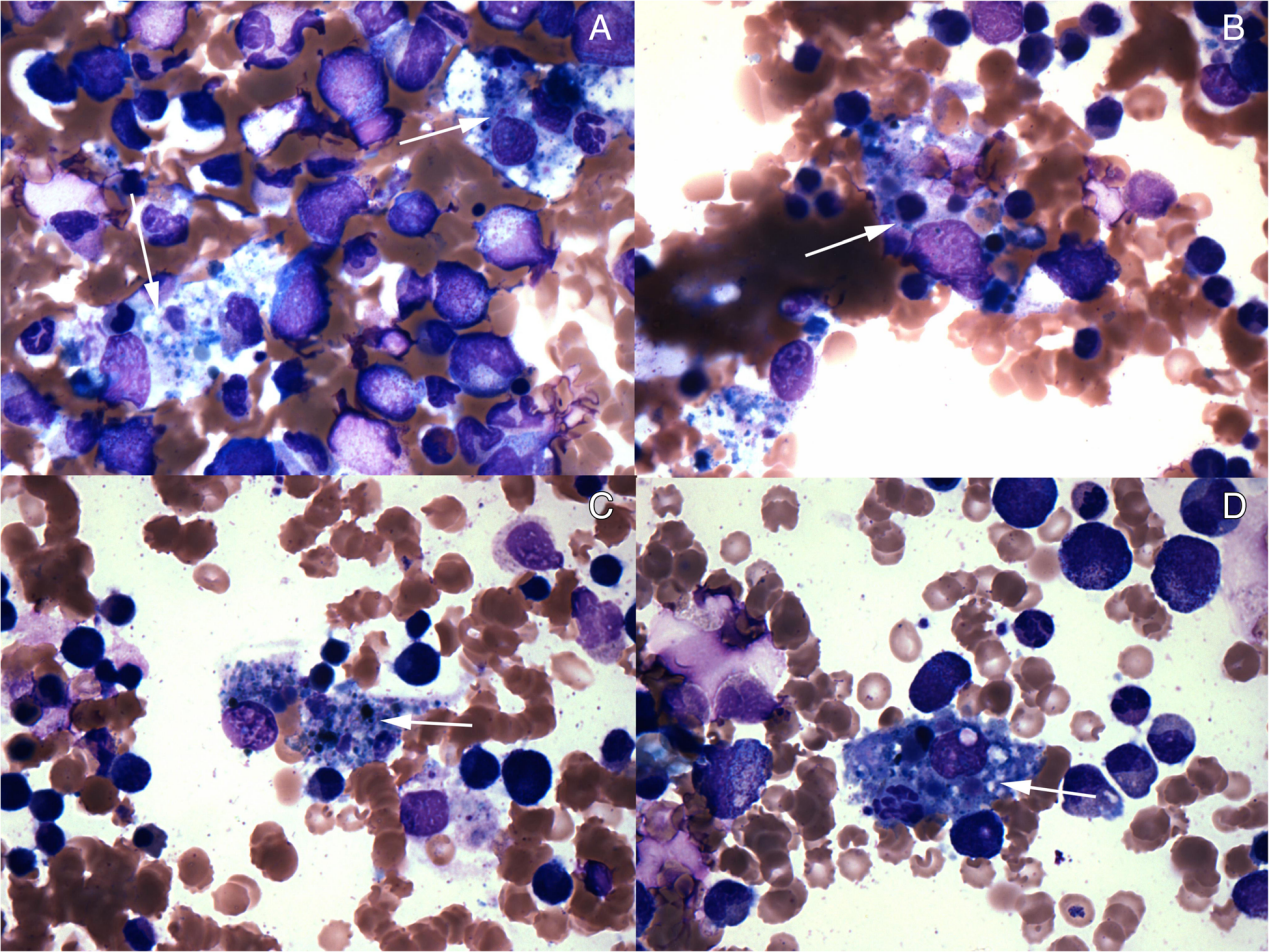
Bone Marrow Biopsy of HLH (**a**-**d**). The marrow showed trilineage hematopoiesis with relative granulocyte hyperplasia (M:E ratio 6.2:1). Erythroid and granulocyte precursors showed orderly maturation with no evidence of dyspoiesis or increased blasts. Megakarycoytes were adequately present and show normal morphology. Lymphocytes and plasma cells were unremarkable. Macrophages were mildly increased with evidence of hemophagocytosis and show frequent forms with ingested mature red cells, erythroid precursors, occasional leukocytes and platelets. Arrows indicate sections associated with hemophagocytosis

Due to the new onset of pancytopenia, a bone marrow biopsy was performed revealing a hypercellular marrow with granulocyte hyperplasia and the presence of hemophagocytosis; suggestive of HLH (Fig. 1). Skin biopsies from the torso showed full-thickness epidermal necrosis with subepidermal blister formation and absence of hemophagocytosis. A second skin biopsy done from the purpuric right fifth toe showed ulceration with focal epidermal and fat necrosis and dermal hemorrhage suggestive of ischemic changes.

He also began to clinically deteriorate with refractory hypotension and tachycardia. At this time, he was suspected to have new onset acute cutaneous SLE presenting with bullous lesions with secondary HLH. Therefore, he was treated with methylprednisolone 1000 mg IV daily for a three-day pulse course and then was switched to prednisone 60 mg daily. He was also started on hydroxychloroquine 400 mg daily. At time of discharge, his blood counts had slightly improved and fevers had dissipated with hemodynamic stability. One month after discharge, he was seen in the outpatient rheumatology clinic with resolution of the skin rash and improving blood counts (hemoglobin 112 g/L, platelets and white blood cell count normal) and ferritin of 1065 μg/L.

## Case presentation

A 43-year-old African-Canadian male presented with a 6-week history of weight loss of 20 pounds, fatigue, persistent low-grade fever and a prominent malar rash and extensive diffuse desquamative rash on most of the body. On further history, he noted arthralgia for the preceding 20 years and a similar intermittent rash. Past medical history was non-contributory and he denied any medication use. Review of systems was otherwise unremarkable.

Initial laboratory investigations showed a complete blood count that was significant for hemoglobin 84 g/L, platelets of 80 × 10^9^/L, alanine aminotransferase at 145 U/L, fibrinogen of 110 mg/dL and creatinine of 145 μmmol/L. He was subsequently admitted under the general internal medicine team for management of acute kidney injury.

Further bloodwork showed a positive ANA of 1:640 with a speckled morphology, anti-Ds DNA intermediate at 9 kIU/L and ENA with moderately positive U1-RNP, high positive Smith antigen, low positive Scl-70 and SS-A/Ro 60 moderately positive. Decreased C3 of 0.15 g/L, C4 of 0.02 g/L, and ferritin of 2989 μg/L were also observed. He was treated with methylprednisolone 1000 mg IV daily for a three-day pulse course. The clinical course deteriorated with anuric renal failure requiring hemodialysis and hypoxic respiratory failure secondary to pneumonia precipitating ICU admission and intubation. Bloodwork done at that time showed ferritin level of greater than 100,000 μg/L, decreased fibrinogen of 1.5 g/L and elevated triglycerides of 5.5 mmol/L; in keeping with development of HLH secondary to SLE. Renal biopsy in the context of anuria showed lupus nephritis, class I with acute tubular injury. The patient required multiple courses of methylprednisolone IV then switched to oral prednisone, anakinra, cyclophosphamide IV and hydroxychloroquine. Eventually, his clinical course improved with ability to be extubated and subsequently discharged. At time of discharge, his blood counts had improved and the cutaneous rash had dissipated. He continues to be followed in the outpatient rheumatology setting and disease has been quiescent on mycophenolate mofetil 1 g twice daily, anakinra 100 mg subcutaneous daily, and hydroxychloroquine 400 mg daily. This case represents a delayed diagnosis of SLE progressing to severe skin manifestation of the disease complicated with secondary HLH (Table 1).

**Table 1:** Diagnostic Criteria of HLH as defined by the HLH-2004 Criteria

HLH Criteria	Case 1	Case 2
Fever ≥38.5 °C	+	+
Splenomegaly	–	–
Hemoglobin < 9 g/dL^a^	–	+
Platelets < 100,000 microL^a^	+	+
Absolute Neutrophil Count < 1000/microL^a^	–	–
Hypertriglyceridemia: Fasting Triglycerides > 265 mg/dL	+	+
Hypofibrinogenemia: Fibrinogen < 150 mg/dL	–	+
Hemophagocytosis in bone marrow, spleen, lymph node or liver	+	–
Low or absent NK cell activity	n/a	n/a
Ferritin > 500 ng/mL	+	+
Elevated soluble CD25 (soluble IL-2 receptor alpha) two standard deviations above age-adjusted laboratory-specific norms	+	–

^a^ Peripheral blood cytopenia as one criteria

## Discussion and conclusions

HLH is a rare but devastating clinical entity and has been associated with several rheumatologic disorders including adult Still’s disease, sarcoidosis, systemic sclerosis and Sjogren’s syndrome [7]. An extensive literature review was completed for articles published up to September 2017 based on a bibliographic search in the PubMed database using the keywords “Hemophagocytic lymphohistiocytosis” and “Systemic lupus erythematosus” with inclusion criteria of articles focusing on cutaneous manifestation. Case reports involving new diagnosis of SLE as the onset of HLH is limited [8–12]. In the cases presented here, case 1 and 2’s clinical onset of HLH did coincide with the new onset of SLE and does fulfill the SLICC criteria for SLE. Amongst all cases of SLE and HLH, including rheumatologic flares, the incidence of HLH is approximately 0.9–4.6% [13]. As has been described by several groups, the difficulty in making the diagnosis of HLH in new onset SLE arise from the overlap of many symptoms, making the use of the HLH-2004 criteria critical for acute and timely diagnosis. Several other parameters have been used to tease out the elusive diagnosis, however with mixed consensus in the literature. Hyperferritinemia has been cited as the best parameter to distinguish between active SLE flare and HLH-associated SLE with a sensitivity and specificity of nearly 100% [1]. However, hyperferritinemia in the HLH-94 study indicated that in the pediatric population, ferritin level > 500 mcg/L was 100% sensitive for HLH but less specific; whereas ferritin > 10,000 mcg/L was 90% sensitive and 96% specific for HLH [1]. In the adult population, the correlation between hyperferritinemia and HLH is less clear, particularly with the presence of an overlapping autoimmune condition such as SLE. One study evaluated HLH-associated SLE with solely active SLE and concluded a high ferritin level often points towards the former [7].

More recently, a group from France has published the “HScore”, a well-validated scoring system for the diagnosis of HLH [1]. The scoring system incorporates parameters including organomegaly, ferritin, ALT, degree of cytopenia, fibrinogen, fever, and hemophagocytosis with a HScore > 250 conferring 99% probability of HLH and a score < 90 at < 1% probability. Interestingly, for case 1 the HScore was calculated as 245 (conferring 99.1% probability of HLH) and case 2 as 201 (conferring 88.8% probability for HLH). In the future, the HScore may be a useful clinical parameter to help tease out active SLE from HLH-associated SLE.

The two cases here highlight an extremely rare entity within HLH-associated SLE with severe cutaneous manifestation at the time of diagnosis. A case report from Japan also observed this entity with erythematous plaques as the initial manifestation of HLH in a patient with newly diagnosed lupus [14]. Furthermore, HLH can present with skin manifestation but (in) the absence of antibodies can eliminate an associated autoimmune process.

In patients with known SLE, the parameters to gauge flare from HLH is even more difficult. One study assessed patients with known autoimmune conditions (including SLE) and performed skin biopsies on three patients during acute flare, of which all were found to have hemophagocytosis [15]. While this may be an important additional clue to help tease apart the two conditions, hemophagocytosis is a non-specific finding and has been associated with self-limiting bone marrow infections in the context of activated macrophages. A laboratory study assessed hemophagocytosis by comparing bone marrow aspirates in patients with known HLH compared to random control bone marrows [16]. The sensitivity of hemophagocytosis in HLH was 83% with a specificity of only 60%, suggesting rare hemophagocytes may be seen in normal bone marrow. The authors suggest a rise of hemophagocytosis count threshold to increase to 0.05–0.13% to account for rare ‘normal’ markers would increase specificity to 100%, which may also aid in teasing out HLH-associated SLE from SLE flare.

Amongst all currently available immunologic studies available, sIL-2R appears to correlate best with disease activity. One study assessed patients with lymphoma-associated HLH compared to non-lymphoma cases and found that the former had a much higher sIL-2R to ferritin ratio (8.56 vs. 0.66) [17]. These ratios have not been assessed in autoimmune associated HLH, specifically SLE, and may provide a promising avenue to better discern this diagnosis.

Classically, treatment of primary HLH is directed towards use of the HLH-2004 protocol including etoposide, dexamethasone, cyclosporine, consideration of intrathecal methotrexate and finally with hematopoietic stem cell transplantation [18]. Treatment of secondary HLH is less clear, but ultimately relies on treatment of the primary auto-immune disease. In the two cases presented, SLE was the process associated with HLH and corticosteroids were rapidly initiated, in keeping with previously described cases [19]. Because of the high mortality risk of HLH, the absence of rapid improvement of symptoms, even in the context of non-severe manifestations of SLE, requires aggressive immunosuppression with cyclophosphamide and frequently the addition of biologics such as Anakinra (Il1 inhibitor), infliximab (TNF inhibitor) [20] and alemtuzumab (CD52 inhibitor) [6, 21, 22].

Based on the cases and literature review presented here, we suggest considering HLH in the differential diagnosis with adult patients presenting with new onset, severe cutaneous manifestation of lupus and to consider appropriate investigations and prompt treatment.

## Abbreviations

ANA: Anti-nuclear antibody
ANCA: Anti-neutrophil cytoplasmic antibody
CRP: C-reactive protein
ENA: Extractable nuclear antigen
HLH: Hemophagocytic lymphohistiocytosis
IL-2: Interleukin-2
MPO-ANCA: Myeloperoxidase-antineutrophil cytoplasmic antibody
SLE: Systemic lupus erythematosus
